# Design Mechanism and Property of the Novel Fluorescent Probes for the Identification of *Microthrix parvicella* In Situ

**DOI:** 10.3390/ma10070804

**Published:** 2017-07-15

**Authors:** Xiumei Jiao, Xuening Fei, Songya Li, Dayong Lin, Huaji Ma, Baolian Zhang

**Affiliations:** 1 School of Environmental Science and Engineering, Tianjin University, Tianjin 300072, China; jiaoxiumei@163.com (X.J.); lsongya90@163.com (S.L.); wjlinday@163.com (D.L.); 2 Tianjin Key Laboratory of Aquatic Science and Technology, School of Environmental and Municipal Engineering, Tianjin Chengjian University, Tianjin 300384, China; mahjh510@163.com; 3 School of Science, Tianjin Chengjian University, Tianjin 300384, China; ybysw@126.com

**Keywords:** fluorescent probes, sludge bulking, identification, *Microthrix parvicella*, quantitative analysis

## Abstract

In this study, two novel fluorescent probes, probe A and probe B were designed, synthesized and characterized, based on *Microthrix parvicella* (*M. parvicella*) preferring to utilize long-chain fatty acid (LCFA), for the labeling of *M. parvicella* in activated sludge. The molecular structure of probe A and probe B include long-chain alkane and LCFA, respectively. The results indicated that probe A and probe B had a large stokes shift of 118 nm and 120 nm and high quantum yield of 0.1043 and 0.1058, respectively, which were significantly helpful for the fluorescent labeling. As probe A was more stable than probe B in activated sludge, and the fluorescence intensity keep stable during 24 h, probe A was more suitable for labeling *M. parvicella* in situ. In addition, through the Image Pro Plus 6 (IPP 6) analysis, a quantitative relationship was established between sludge volume index (SVI) and integral optical density (IOD) of the labeled *M. parvicella* in activated sludge samples. The relationship between IOD and SVI conforms to Logistic curve (*R*^2^ = 0.94).

## 1. Introduction

Sludge bulking is a frequently occurring phenomenon in wastewater treatment plants (WWTP) [1,2], which can cause serious operational problems. It has been reported that sludge bulking mainly results from the excess growth of filamentous bacteria [3,4]. The heterogeneous structure of activated sludge flocs consists of a variety of microorganisms as well as organic and inorganic particles, and dead cells surrounded by extracellular polymeric substances [5]. In the ideal condition of balancing the number of filamentous and floc-forming bacteria, they can combine into large irregular flocs to create activated sludge. Besides serving as the filamentous backbone of microbial flocs, filamentous bacteria can excessively grow outside the flocs and protrude into the liquid, which bring about the filamentous bulking. The floc structure in this case is observed as more diffuse and floc-to-floc bridging can occur more frequently. Thus it can be used to determine the degree of sludge bulking by study on the change of the structure and the number of filamentous bacteria inside and outside the sludge flocs. A large number of national surveys have shown that *Microthrix parvicella* (*M. parvicella*) is frequently responsible for causing problems concerning solid-liquid separation in bulking [6,7,8].

The conventional method of researching *M. parvicella* based on sludge volume index (SVI) and morphological features [9,10] can make a preliminary decision on sludge bulking according to the SVI and the typical characteristics of *M. parvicella* showed by the classical Gram staining and Neisser staining [9]. When this method was applied in determining the abundance of the bacteria, experienced staff was required because of morphological variability resulting from different growth conditions [11]. Molecular biology technique is another excellent method which can avoid the problems of studying microbial populations without relying on traditional methodology. The techniques of fluorescence in situ hybridization (FISH) and polymerase chain reaction (PCR) have been widely applied to the identification study of *M. parvicella* [11,12].

FISH with rRNA-targeted probes is a staining technique that allows phylogenetic identification of bacteria in mixed assemblages without prior cultivation. The method is mainly based on identifying the rapidly increasing set of bacterial small subunit (16S rRNA) rRNA sequences. In theory, each ribosome can be stained by one probe molecule during the hybridization procedure. Some FISH probes target at *M. parvicella* have been designed and evaluated for the detection and identification of the bacteria in situ by Erhart et al. [13], which provides an effective tool for the research of *M. parvicella*. The use of oligonucleotide probes identifying 16S rRNA presents a revolution in identification of *M. parvicella* both for basic research and practical application. It was stated that even though FISH improves biological processes, its time consuming and laborious process can be one of the disadvantages [14,15]. Therefore, a new method to identify in situ and quantify the *M. parvicella* rapidly is necessary and urgent.

It has been shown by microsphere adhesion-to-cell (MAC) measurements that the surface of *M. parvicella* appears to be more hydrophobic than the most of other bacteria in activated sludge [16]. *M. parvicella* can adsorb and metabolize long chain fatty acids (LCFA) under aerobic and anaerobic conditions [17,18,19]. McIlroy et al. [20] concluded the metabolic mechanism of LCFA. Firstly, the LCFAs were absorbed to the surface of *M. parvicella* before transporting through the exopolymeric layer into the cell, and finally were metabolized by *M. parvicella*.

The fluorescent dye has a number of advantages. For instance, large emission wavelength can avoid the fluorescence background interference of microorganisms themselves. Wide spectral range, large molar absorptivity, high quantum yield can improve the sensitivity of identification and reduce low detection limit. Many fluorescent molecule dyes were developed, especially the cyanine dyes, which has been widely applied in environmental microbiology studies [21,22]. Carbazole cyanine dyes and the derivatives with the nitrogen heterocyclic structure possess the advantages of large rigid plane conjugate system, large stokes shift, high fluorescence intensity and considerable stability [22,23,24].

**Figure 1:**
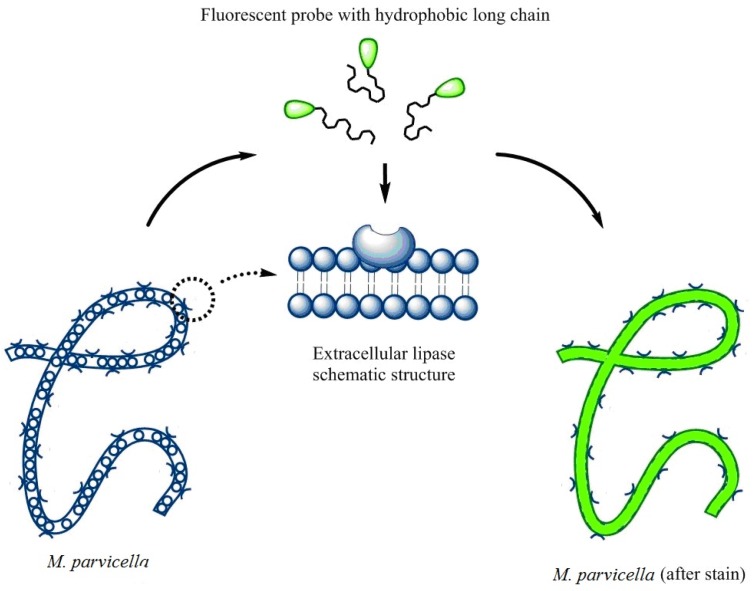
A schematic explaining the potential interactions between the fluorescent probe and *M. parvicella* filaments.

The objective of this study was to achieve real-time identification of living *M. parvicella* in situ activated sludge by fluorescent probes modified by hydrophobic long-chain alkane or LCFA. According to the characteristics of *M. parvicella* feeding on LCFA, we managed to identify *M. parvicella* in the activated sludge samples. In order to achieve high fluorescence intensity, high quantum yield and large stokes shift, a series of modified carbazole cyanine dyes integrating long-chain alkane and LCFA were designed and synthesized. The probes were used to identify *M. parvicella* in situ. Biological stability of the probes for identifying *M. parvicella* in situ was tested. In addition, the Image Pro Plus 6 (IPP 6) processing software was used to establish the intensity of fluorescence probe with SVI quantitative relationship. The fluorescent probes in this study can be absorbed preferentially by *M. parvicella* in activated sludge samples (the schematic reaction between the probes and *M. parvicella* was shown in the Figure 1).

## 2. Materials and Methods

### 2.1. Activated Sludge Samples

A few activated sludge samples with abundant growth of *M. parvicella* were collected from an activated sludge wastewater treatment plant (WWTP) in Tianjin (China). The sludge concentration was between 3–6 g SS/L (SS, Suspended Solid). The labeling campaign took place between September, 2015 and May, 2016. The activated sludge sample was collected from the secondary sedimentation tanks inlet of the WWTP in Tianjin on the day before the identifying experiments. Those sludge samples for the experiment of labeling *M. parvicella* were stored at 4 °C. Before using, the sludge was diluted to appropriate concentrations with supernatant from the activated sludge samples.

### 2.2. Synthesis

**Figure 2:**
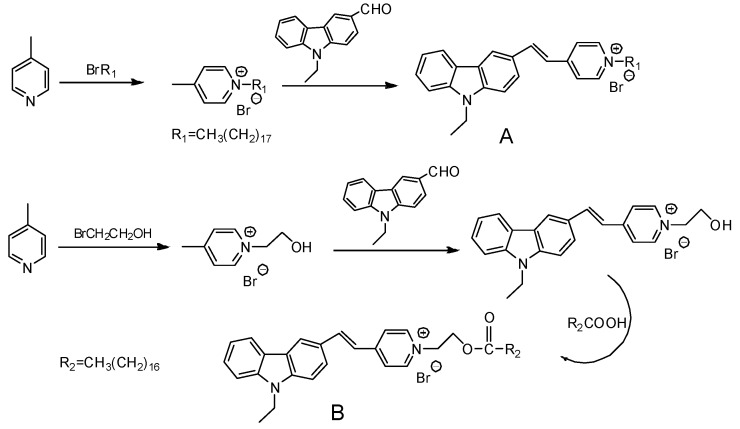
Synthesis of probe A and probe B.

The synthesis procedures of the novel probe A and probe B are shown in Figure 2.

The synthesis procedure of compound N-Ethyl-carbazolyl-3-aldehyde (structure shown in Figure 2) was based on the reported [25]. 4-Methylpyridinium Bromide (10 mmol), N-Ethyl-carbazolyl-3-aldehyde (10 mmol) and piperidine (1.0 mL) were dissolved in 25 mL ethanol (EtOH). The mixture was stirred overnight. The solvent was removed to get the yellow precipitation. After filtering, the crude product was purified by silica-gel column chromatography to obtain probe A.

The typical procedure of compound B was described as follows. The mixture of 4-methyl pyridine and bromoethanol were reacted in toluene to produce salt at 120. Then the salt (10 mmol), N-ethyl-carbazolyl-3-aldehyde (10 mmol) and some drops of catalytic piperidine were added to the flask with 25 mL ethanol (EtOH). The mixture was stirred overnight at 80 °C to obtain orange precipitation. The mixture of the orange precipitation (1 mmol), stearic acid (1 mmol), 1-(3-Dimethylaminopropyl)-3-ethylcarbodiimide hydrochloride (EDCl) (1.2 mmol) and 4-dimethylaminopyridine (DMAP) (0.2 mmol) in 20 mL dichloromethane (DCM) were mixed and stirred overnight at room temperature. After reaction, the organic solvent was removed by rotary evaporation. The residue was purified by silica-gel column chromatography to get probe B.

### 2.3. Spectral Properties

#### 2.3.1. Absorption and Fluorescence Measurements

The UV/Vis absorption spectra and fluorescence spectra were scanned on a Perkin Elmer LAMBDA 35 spectrometer (PerkinElmer, Waltham, MA, USA) and a Hitachi F-7000 (Hitachi, Tokyo, Japan) respectively. ^1^H NMR spectra were obtained on a Bruker (400 MHz) spectrometer (Bruker, Karlsruhe, Germany). The excitation and emission slits were 5 nm and the PTM voltage of the spectrometer were 400 V.

The absorption and fluorescence spectra of probe A and probe B were measured in methanol (MeOH), EtOH and DCM, dimethyl formamide (DMF), and dimethyl sulfoxide (DMSO) respectively.

#### 2.3.2. Fluorescence Qzantum Yields

Fluorescence quantum yields of the compounds were determined according to the following equation using fluorescein in NaOH (0.1 mol/L) aqueous solution with a fluorescence quantum yield of 0.90 [26] to calculate relative fluorescence quantum yields:

(1) $$\varphi_{x}=\varphi_{s}\cdot \frac{F_{x}}{F_{s}}\cdot \frac{A_{s}}{A_{x}}\cdot {\left(\frac{n_{x}}{n_{s}}\right)}^{2},$$

where *φ_x_* and *φ_s_*are the fluorescence quantum yield of the sample and reference respectively, *F_x_* and *F_s_* are the integrated fluorescence spectra for the sample and reference, respectively, *A_x_* and *A_s_* are the absorbance for the sample and reference at the excitation wavelength, respectively, and *n_x_* and *n_s_* are the refractive indexes of the sample and reference, respectively.

### 2.4. Photostability

The photostability of fluorescent probes has attracted a great attention of experts, because it plays an important role for its application as biological fluorescent labels. In this study, we used iodine-tungsten lamp (500 W) as the illuminant. To decrease the effect on the stability caused by the heat from illumination, we used the NaNO_2_ aqueous solution (50 g/L) as the cold trap. The experiment was performed in EtOH at room temperature. The absorption and fluorescence intensity of probe A and probe B was measured after exposing to light with different time.

### 2.5. The Labeling of M. parvicella

Appropriate doses of probe A or probe B EtOH solution (1.0 mmol/L) were added to the activated sludge samples (10 mL) and shaken for 5 min at room temperature. The effect on labeling *M. parvecella* was investigated by fluorescent inverted microscope (OLYMPUS-IX71 (Olympus, Tokyo, Japan), and images were obtained (×400).

For investigating the effect of concentration of fluorescent probe on identifying *M. parvicella*, labeling tests in probe concentration of 0.0025, 0.005, 0.0075, 0.01, 0.015, and 0.02 mmol/L were performed for sludge samples. According to the testing method described above, 15 randomly chosen images were taken for avoiding biases [27]. The images were analyzed by the IPP 6 software (6.0, Media Cybernetics, Rockville, MD, USA). We also investigated the effect of the different duration on the labeling *M. parvecella* through analyze the images by the IPP 6.

### 2.6. Images Analysis

All captured images were analyzed by the IPP 6. The process of obtaining the IOD value and Area value was accorded to the reported previously [28]. In this study, the IOD is the sum of the fluorescence intensity of every *M. parvicella* filament of the images. It can be considered as the number of *M. parvicella* in the examined sludge samples. The Area is the pixel of the extracted *M. parvicella* by the IPP 6. The Area and IOD of each image can be obtained through analyzing the images, and the mean fluorescence intensity can be calculated by IOD/Area. The average IOD/Area in every condition can be obtained, as the mean fluorescence intensity.

To assess the association of the fluorescence intensity of *M. parvicella* with the SVI of activated sludge, probe A was used to label the activated sludge samples of various SVI. To avoid random errors, 15 pictures of each sample were randomly captured and processed by IPP 6. Then, the average of the IOD was taken as the number of *M. parvicella* in corresponding samples.

## 3. Results

### 3.1. ^1^H NHM of the Fluorescent Probes

To improve the sensitivity of fluorescent probe, reduce its detection limit, and not to interfere to the morphology of *M. parvicella*, we designed the fluorescent probes with high fluorescence intensity and quantum yield by molecular structure optimization on the basis of previous study.

The ^1^H NMR spectra results and mass spectral analyses of the fluorescent probe A and probe B are shown below. The results show that the molecular structures of probe A and probe B were correct.

Probe A (400 MHz, CDCl_3_), δ (ppm): 8.91 (d, *J* = 3.0 Hz, 2H), 8.34 (s, 1H), 8.16–8.26 (m, 4H), 7.94 (d, *J* = 4.8 Hz, 1H), 7.76 (d, *J* = 4.2 Hz, 1H), 7.68 (d, *J* = 4.2 Hz, 1H), 7.50–7.59 (m, 2H), 7.33 (t, *J* = 7.6 Hz, 1H), 4.43–4.50 (m, 4H), 1.89–1.92 (m, 2H), 1.37 (t, *J* = 7.0 Hz, 3H), 1.18–1.30 (m, 30H), 0.84 (t, *J* = 6.8 Hz,3H). ESI-TOF-MS calculated for C_39_H_55_N_2_^+^: 551.4360; Found: 551.4182.

Probe B (400 MHz, CDCl3), δ (ppm): 9.11 (s, 2H), 8.33 (s, 1H), 8.14 (d, *J* = 3.8 Hz, 1H), 7.92 (t, *J* = 16.4 Hz, 3H), 7.76 (d, *J* = 4.2 Hz, 1H), 7.54 (t, *J* = 7.8 Hz, 1H), 7.42 (t, *J* = 8.4 Hz, 2H), 7.32 (t, *J* = 7.4 Hz, 1H), 7.16 (d, *J* = 8.2 Hz, 1H), 5.22 (s, 2H), 4.46 (s, 2H), 4.34 (q, *J* = 10.6 Hz, 2H), 2.31 (t, *J* = 7.0 Hz, 2H), 1.56 (s, 2H), 1.46 (t, *J* = 7.0 Hz, 3H), 1.15–1.35(m, 28H), 0.84 (t, *J* = 6.8 Hz, 3H). LC-Q TOF-MS calculated for C_41_H_57_N_2_O_2_^+^: 609.4415; Found: 609.4419.

### 3.2. Spectral Properties of Fluorescent Probe A and Probe B in Different Solvents

Solvation effects on the fluorescent probe was prone to be affected by the properties of organic solvents such as dipole moment, dielectric constant and polarity, so spectral properties of the maximum absorption wavelength and emission wavelength were clearly observed.

**Figure 3:**
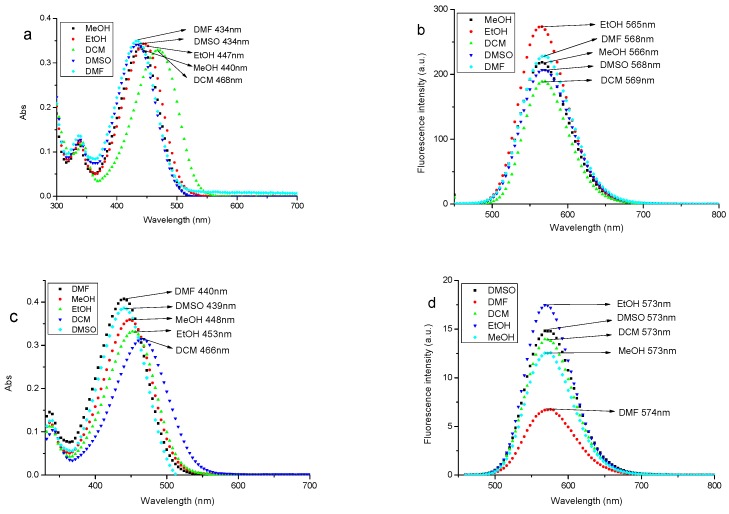
Spectra of the fluorescent probe A and probe B in MeOH, EtOH, DCM, DMF, and DMSO solvents, respectively (UV-Vis absorbance spectra of probe A (**a**) and probe B (**c**); and fluorescence emission spectra of probe A (**b**); and probe B (**d**)).

The spectra are shown in Figure 3 and parameters of solvents are summarized in Table 1.

As shown in Figure 3, the maximum absorption wavelength (*λ*_max_) of probe A and probe B in different solvents were around 440 nm. The maximum absorption wavelength of probe A and probe B in different solvents all present DMSO < DMF < MeOH < EtOH < DCM, and the maximum absorption wavelength of probe A and probe B are all red-shifted(Figure 3a,c). In DCM, *λ*_max_ of probe A and probe B were the biggest and located at 468 nm and 466 nm respectively. The spectral data of probe A and probe B in different solvents are presented in Table 1. The orders of magnitude of molar extinction coefficient are up to 10^4^, and probe A and probe B both show large stokes shifts, 101–134 nm and 107–134 nm, respectively. Table 1 shows that stokes shifts are also affected by different solvents, and stokes shifts of probe A and probe B have the regular change; more stokes shifts with greater polarity of the solvents. The results suggest the new fluorescent probe A and probe B in this study had effective and tangible spectral characteristics. We chose EtOH as the solvent to dissolve the probe stock solution in the experiment of labeling *M. parvicella*, because the fluorescence intensity of probe A and probe B were higher than in other solvents.

### 3.3. The Spectral Characteristics and Structure-Function Relationship of Organic Fluorescent Probes

To analyze the influence of molecular structure changes on spectral characteristics, the R group in the molecular probes in Table 2 was replaced by different long-chain alkane. Thus a series of modified fluorescent probes were synthesized according to the synthesis method of probe A as shown in Figure 2. To investigate the different spectral properties with respect to probes in the previous study [28], we compared the spectral characteristics of these probes in EtOH with the study, and the results are shown in Table 2.

Table 2 shows the spectral characteristics of the probe of series 1 and series 2, as *λ*_max_, *ε*, *λ*_em_ and Stokes shift. Due to no strong conjugated structure between alkyl chain and carbazole group, the maximum absorption wavelength (*λ*_max_) Stokes shift, molar absorptivity concomitant (*ε*) and maximum emission wavelength (*λ*_em_) were not affected by the different alkyl chain. The Table 2 also shows, the fluorescence intensity of the probes of series 1 is ranging from 17.43 to 296.2 in the ethanol as the solvent. For the two series of probes, the two compounds have the higher fluorescence intensity when the modification is CH_3_(CH_2_)_11_ with the number of 296.2 and 35.98, respectively. We can also see in Table 2 that the changes of organic fluorescent molecule matrix obviously influence the quantum yield of probes. In the fluorescent probe molecules, when carbazole was connected with quinoline by the bridged bond, the probes had larger fluorescence intensity, due to retain the structure of indole of the segment of CY3 in the carbzole molecules. However, when the quinolone group was replaced by the pyridine, the new probe was formed with higher quantum yield than the original probe molecules an order of magnitude (such as CH_3_(CH_2_)_15_ modified fluorescent probes, the quantum yield increased from 0.0127 to 0.1036). Thus, the new probe molecules had more effective spectral characteristics and higher detection sensitivity. We tested the new probe on labeling *M. parvicella* in the activated sludge, and the result showed that *M. parvicella* can be highlighted apparently by the probe (Figure S1). Therefore, the bacteria were distinguished from the other parts of the sludge flocs.

### 3.4. Photostability

The photostability of fluorescent probe takes an important part in the labeling, so it can be necessary to investigate the photostaility of probes. The bar chart between the intensities and the exposure time are shown in Figure S2. Figure S2a,b shows that the changes of both absorption and fluorescence intensity are no prominent for both probe A and probe B. The result indicated that probe A and probe B can still keep the intensity after long light exposure which is beneficial labeling *M. parvicella*.

### 3.5. The Labeling of M. Parvicella

#### 3.5.1. Determination of the Optimal Identification Concentration

The results of spectral characteristics suggest that the probe A possesses the highest fluorescence intensity and quantum yield, so the probe A was used to identify the *M. parvicella* in situ activated sludge. Whether *M. parvicella* was in the internal of flocs or protruding from the flocs can be extracted after the imagesare processed by IPP 6 (Figure S3). Then, the Area and IOD of the extracted filaments were counted by IPP 6.

**Figure 4:**
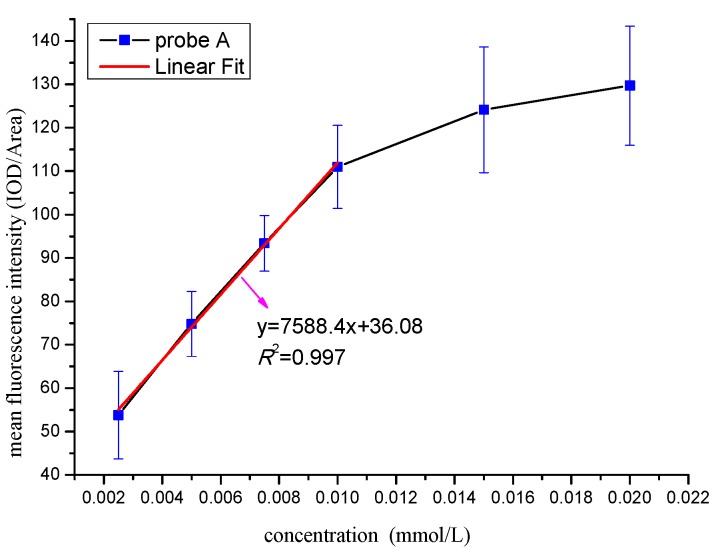
Effect of different concentration of probe A on the mean fluorescence intensity.

To determine the optimal identification concentration of the probe A, we set up the curve between the mean fluorescence intensity and concentration (Figure 4). According to Figure 4, there is an increasing trend of the mean fluorescence intensity when the concentration is less than 0.01 mmol/L, and it presents the obvious linear relationship (*R*^2^ = 0.997) between the intensity and the concentration. When the concentration exceeded 0.01 mmol/L, there is also an increasing trend, but the linear relationship becomes negative. The reason is that when the concentration of probe is too high, not only the *M. parvicella* can absorb the fluorescent probe, sludge flocs can also absorb a large amount of free probe molecules, which makes the *M. parvicella* very difficult to distinguish from the flocs. Consequently, the best concentration for probe A was 0.01 mmol/L.

#### 3.5.2. Labeling Effect

**Figure 5:**
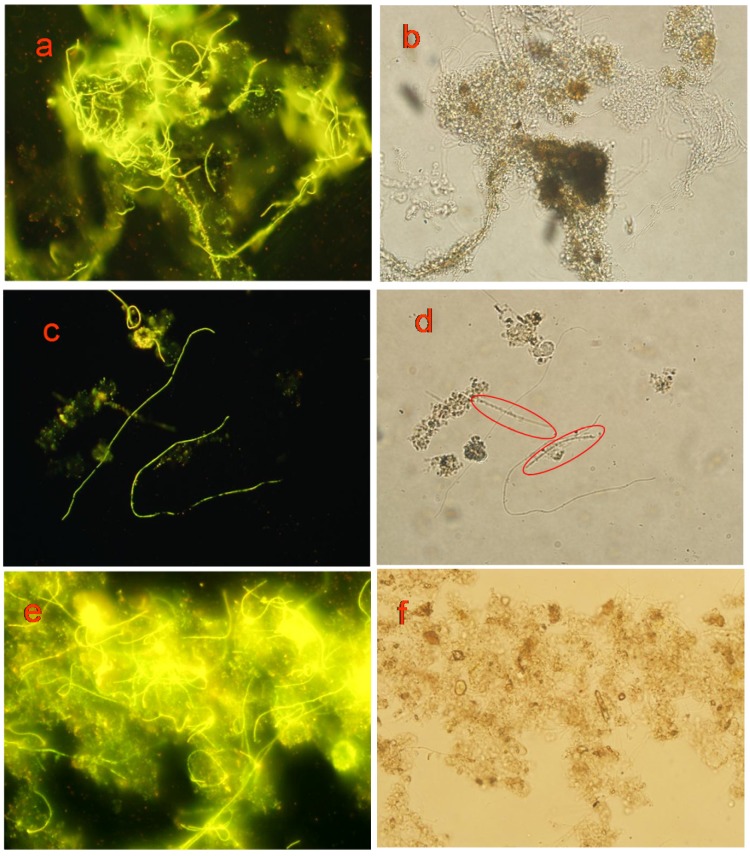
Images of observation under fluorescent light and nature light (400×). The microscope images taken in the labeling of *M. parvicella* by: probe A (**a**,**c**); and probe B (**e**) under fluorescent light; and the images under natural light of: probe A(**b**,**d**); and probe B (**f**).

As shown in Figure 5, probe A and probe B can label *M. parvicella* filaments inside the flocs and protruding from the flocs to show fluorescence. There is no significant difference in identifying effect of *M. parvicella* in situ, and all the filaments have a bright yellow-green fluorescence (Figure 5a,c,e). Comparing Figure 5c,d, it is evident that some filaments (labeled by red circles in Figure 5d) can be observed in natural lightthat are not labeled by the fluorescent probe (Figure 5c), which can differentiate *M. parvicella* from other filaments. Thus, probe A and probe B could effectively label *M. parvicella* in the activated sludge.

#### 3.5.3. Biological Stability of the Probe

**Figure 6:**
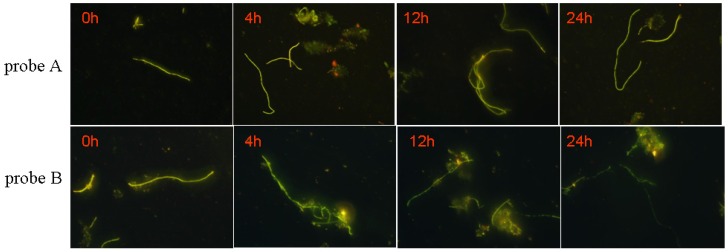
Images of the probes A and B duration time in labeling.

**Figure 7:**
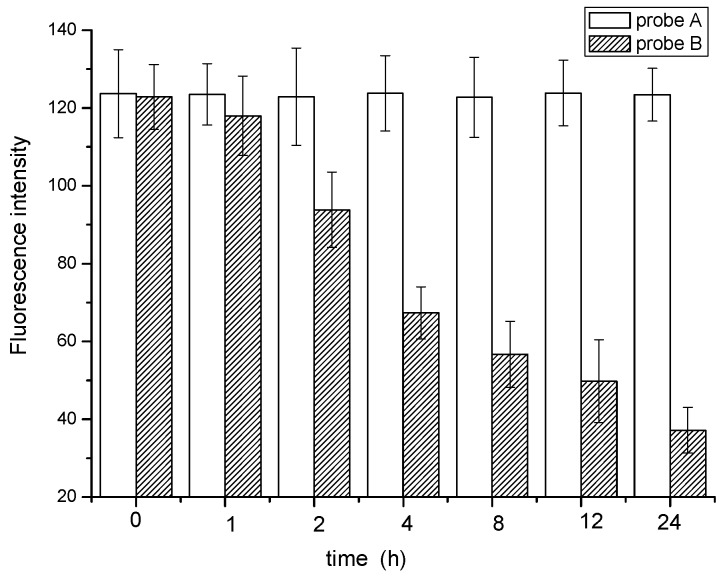
The mean fluorescence intensity of different duration time.

Biological stability of probe plays an important role in identification because the structure of probe is prone to be decomposed by the microorganisms in sludge. The result is shown in Figure 6 and Figure 7.

Obvious differences can be observed from the identification images of *M. parvicella* for Probe A and probe B. Compared with probe B, probe A appears to have more stable fluorescence intensity when these two probes were both applied to identify the *M. parvicella* for the test time (see Figure 6). The mean fluorescence intensity of probe A did not change tremendously within 24 h, while that of probe B decreased with different durations. The results of quantitative calculation parallel the same trend of the mean fluorescence intensity for probe A and B (see Figure 7).

### 3.6. Quantification of the Fluorescence Intensity and SVI

**Figure 8:**
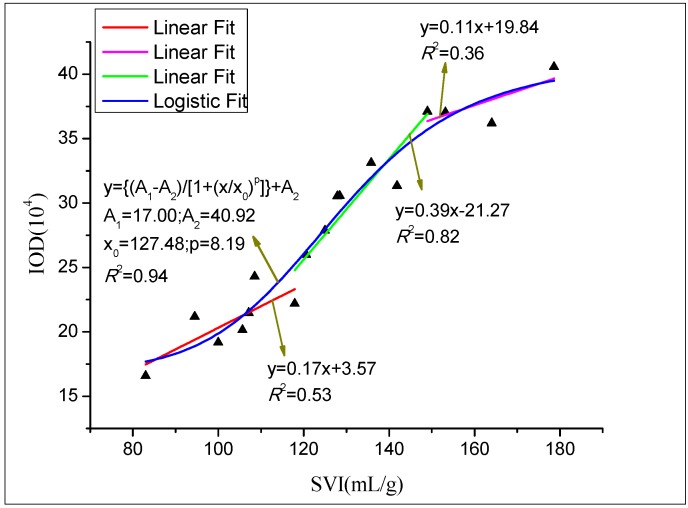
Quantification relationship between SVI and integral optical intensity (IOD).

To determine the relationship between SVI and *M. parvicella* abundance (represented with IOD value), probe A was used to label the activated sludge samples collected from the secondary sedimentation tank of a WWTP between September 2015 and May 2016. The mean IOD of each sample was obtained by the IPP 6 process, and the results are shown in Figure 8. The relationship between IOD and SVI conforms to Logistic curve $y=\frac{A_{1}-A_{2}}{1+{\left(X/X_{0}\right)}^{p}}+A_{2}$ (*R*^2^ = 0.94) (A_1_ = 17.00, A_2_ = 40.92, x_0_ = 127.48, p = 8.19) (Figure 8). There is a moderate association (*R*^2^ = 0.53) of IOD with SVI when SVI is between 80 mL/g and 120 mL/g. During this period, IOD increases slowly, and it indicates that there is a small quantity of *M. parvicella* in the activated sludge samples. However, the linear correlation between IOD and SVI is significantly increased (*R*^2^ = 0.82) when SVI ranges from 120 mL/g to 150 mL/g, and, in this period, there is a high IOD increase rate. The possible reason for this is that *M. parvicella* has excessive growth during this period, leading to the increasing number of the bacteria in the activated sludge. However, the proliferation of *M. parvicella* mainly occurs in the internal of flocs but only a little *M. parvicella* protrude from the flocs (Figure 5a, SVI = 128). When SVI exceeds 150 mL/g, the linear correlation becomes lower (*R*^2^ = 0.36), but IOD increases slowly. This indicates that the number of *M. parvicella* does not increase too much.

## 4. Discussion

Fluorescent probe A and probe B modified by CH_3_(CH_2_)_17_ and CH_3_(CH_2_)_16_COOH based on the property of *M. parvicella* utilizing LCFA were designed. The results of spectral properties displayed differences in various solvents. In DCM, the red-shifted of probe A and probe B were largest (Figure 3). The red-shift can be attributed to the introduction of heteroatom and positive charge in fluorescent probes, the interaction force between organic fluorescent dye molecules and the DCM is small in the process of the solvation effect since the DCM is a weak polar aprotic solvent. The solvent with larger polarity could reduce the energy difference between excited state and ground state, which led to a red-shift of the molecule [29,30].

Table 1 indicates that the number of stokes shift were large in all solvents, even up to 134 nm in CMF and CMSO. Stokes shift were mainly influenced by two factors, refractive index and dielectric constant. The effect of solvents on fluorescent spectra could be stated by Lippert equation [31]. Table 1 and Table 2 indicate that the new probe molecules had more effective spectral characteristics and high detection sensitivity.

The correlation between the mean fluorescence intensity and the concentration of probe A is very high (*R*^2^ = 0.997), when the probe concentration is less than or equal to 0.01 mmol/L. The correlation is moderate when the probe concentration greater than 0.01 mmol/L. The reason is that the probe was excessive when the probe concentration greater, too many probe spread in flocs, so that *M. parvicella* hardly distinguished from the flocs. Not all of the labeled *M. parvicella* could be analyzed by IPP 6, e.g. when the mean fluorescence intensity value is less than the real value. Thus, the correlation becomes lower following the probe concentration greater than 0.01 mmol/L. And the best concentration of probe A was 0.01 mmol/L in this study.

Porbe A and probe B can label all *M. parvicella* filamentous in the activated sludge (Figure 5). It may result in the scale of probe A and probe B at molecular level, so they can penetrate inside the sludge flocs and be absorbed by *M. parvicella.* The process is in accordance with *M. parvicella* transport of fatty acids reported by McIlroy [20]. According to Fei [32], the quantum dots probe can only identify *M. parvicella* filaments protruding from the flocs and free ones in the activated sludge, but it cannot label *M. parvicella* inside the flocs. The quantum dots probe is nano level in size, and bigger than the fluorescent molecule probes, which makes it difficult for the quantum dots probe to penetrate the sludge flocs which are composed of bacterial aggregation. Consequently, the quantum dots probe cannot label *M. parvicella* filaments inside the sludge flocs. The novel fluorescent probe A and probe B which were modified by long chain alkane and LCFA with appropriate hydrophobic property, based on *M. parvicella* being hydrophobic and utilizing LCFA, have excellent targeting properties. Thus, the novel probe A and probe B can identify *M. parvicella* in the activated sludge sample, but other filament bacteria cannot be labeled (Figure 5c,d).

FigureS2 indicates probe A and probe B had good photostability. The result indicated that the probes can be exposed to the strong light for a long time and still maintain their intensity. Thus, we regarded that the potostability of probes A and B did not influence their biological stability. Figure 6 and Figure 7 indicated that probe A has more biological stability than probe B. The reason for this phenomenon is that *M. parvicella* cannot consume the probes modified by the long carbon chain compared to the –COOH group, although the probe A was absorbed on the surface of *M. parvicella* filaments. Thus, the molecular structure was not decomposed or deconstructed and the mean fluorescent intensity did not change. However, probe B containing the –COOR group can be hydrolyzed to LCFA, and then metabolized by *M. parvicella*, which was consistent with the physiological property of *M. parvicella*, consuming the LCFA [17,20,33]*.* The decrease of the mean fluorescent intensity was due to the destruction of probe B structure. Furthermore, *M. parvicella* labeled by probe A and probe B still show fluorescence after 24 h, at the same time, the hydrophobicity lipase on the surface of the bacteria still remain activity during the whole process of labeling. In another words, the *M. parvicella* filaments still stay active and can absorb the fluorescent probes. That demonstrates the probe designed in this study was not toxic for *M. parvicella*.

Compared with the advantages of probe A and probe B, FISH technology needs a pretreatment to sludge samples by bacteriolysin to ensure the cell penetration rate of FISH probe. Thus, it can be seen that the identification method of FISH probe cannot maintain *M. parvicella* in living status.

From the quantification relationship of SVI and IOD (Figure 8), we can draw a conclusion that the increase of SVI can probably result in more and more *M. parvicella* protrude from the flocs, and IOD can be applied for evaluation the abundance of *M. parvicella* in the activated sludge samples.

Because the method is simple and rapid for using the novel probes to identify *M. parvicella* in situ, the novel probes can be used for rapid detection of *M. parvicella* in wastewater treatment plant, and can early warn to the sludge bulking. The operators can adjust the process parameters based on the result of the quantification relationship of SVI and IOD, ensuring the normal operation of the process.

## 5. Conclusions

The novel probe A and probe B were synthesized for an in situ study of the change in abundance of *M. parvicella*. Probe A and probe B had large stokes shift and high quantum yield, which were beneficial for the fluorescent labeling. It is worth noting that probe A and probe B can recognize *M. parvicell*a on the cells’ surface, so the process of labeling was simple and rapid. The optimal concentration of both probes was 0.01 mmol/L. Probe A was more stable than probe B in activated sludge, and the fluorescence intensity remained stable over 24 h. The relationship between SVI and IOD conforms to Logistic curve (*R*^2^ = 0.94).

## Figures and Tables

**Table 1:** Spectra data of probe A and probe B in different solutions.

	Parameters	DCM	EtOH	MeOH	DMF	DMSO
	Dipole moment	1.20	1.69	1.69	3.86	3.96
	Dielectric constant	8.9	24.5	32.7	36.7	47.2
A	*λ*_max_/nm	468	447	440	434	434
	*ε*/10^4^ mol·L^−1^·cm^−1^	3.29	3.44	3.35	3.50	3.42
	*λ*_em_/nm	569	565	566	568	568
	fluorescence intensity	189.41	273.59	218.62	228.71	207.52
	Stokes shift/nm	101	118	126	134	134
B	*λ*_max_/nm	466	453	448	440	439
	*ε*/10^4^ mol·L^−1^·cm^−1^	3.15	3.32	3.59	4.07	3.86
	*λ*_em_/nm	573	573	573	574	573
	fluorescence intensity	13.97	17.43	12.59	6.78	14.91
	Stokes shift/nm	107	120	125	134	134

**Table 2:** Probes’ characterization data.

Probes Sructure		*λ*_max_/nm	*ε*/10^4^ mol·L^−1^·cm^−1^	*λ*_em_/nm	Fluorescence Intensity	Stokes Shift */nm	Quantum Yield
The series 1 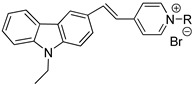 A_1_: R_3_=CH_3_(CH_2_)_7_ A_2_: R_4_=CH_3_(CH_2_)_11_ A_3_: R_5_=CH_3_(CH_2_)_15_ A: R_1_ =CH_3_(CH_2_)_17_ B: 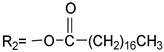	A_1_	445	4.48	563	275.1	118	0.1086
	A_2_	446	4.29	564	296.2	118	0.1051
	A_3_	445	3.35	564	249.2	119	0.1036
	A	447	3.44	565	273.59	118	0.1058
	B	453	3.32	573	17.43	120	0.1043
The series 2 [28] 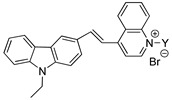 3a:Y_1_= R_3_ 3b:Y_2_= R_4_ 3c:Y_3_= R_5_ 3d:Y_4_= R_1_	3a	502	1.00	620	24.54	118	0.0127
	3b	502	3.83	621	35.98	119	0.0140
	3c	502	2.45	620	27.06	118	0.0127
	3d	502	3.55	620	34.74	118	0.0125

## Supplementary Materials

The following are available online at www.mdpi.com/1996-1944/10/7/804/s1, Figure S1: Images taken in Gram staining (a), FISH staining (b,c) and the novel probe identifying (d) of sludge samples., Figure S2: Photostability of probe A and probe B, Figure S3: Example images analysis for quantification of the mean fluorescence intensity of *M. parvicella*.
