# Supplementary material for: Design Mechanism and Property of the Novel Fluorescent Probes for the Identification of Microthrix parvicella In Situ
Source: Materials (Basel). 2017 Jul 15;10(7):804. doi: 10.3390/ma10070804 (PMC5551847; doi:10.3390/ma10070804)
Supplement: Supplementary file 1 [file materials-10-00804-s001.pdf]

Supplementary Materials: Design Mechanism and Property of the Novel Fluorescent Probes for the Identification of *Microthrix parvicella* in Situ

Xiumei Jiao [1,2], Xuening Fei [1,2,3,*], Songya Li [1], Dayong Lin [1], Huaji Ma [2] and Baolian Zhang [3]

1 School of Environmental Science and Engineering, Tianjin University, Tianjin 300072, China; jiaoxiumei@163.com (X.J.); lsongya90@163.com (S.L.); wjlinday@163.com (D.L.)
2 Tianjin Key Laboratory of Aquatic Science and Technology, School of Environmental and Municipal Engineering, Tianjin Chengjian University, Tianjin 300384, China; mahjh510@163.com
3 School of Science, Tianjin Chengjian University, Tianjin 300384, China; ybysw@126.com
* Correspondence: xueningfei@126.com; Tel.: +86-22-2377-3360; Fax: +86-22-2308-5032

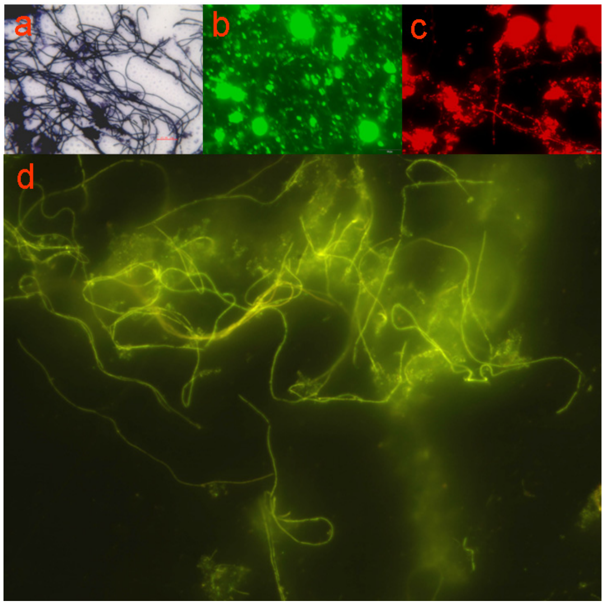

Figure S1. Images taken in Gram staining (**a**), FISH staining (**b**, **c**) and the novel probe identifying (**d**) of sludge samples. Green colour represents for the bacteria encountered in the sludge (EUB mix (EUB338, EUB338II, EUB338III) (**b**); Red color marks the targeted filamentous organism of *M. parvicella* (MPA60, MPA223, MPA645) (**c**); the identification image of novel probe A in our article for *M. parvicella* in situ (**d**). To get the fluorescent images, a 40× objective was used.

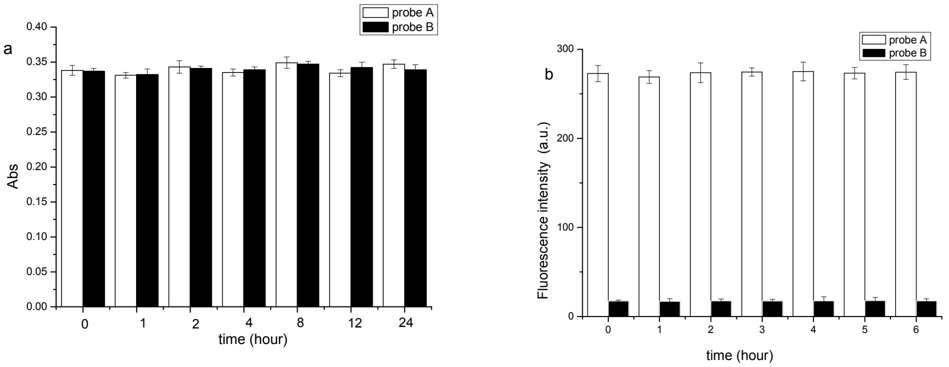

Figure S2 Photostability of probe A and probe B, S2a is the absorption of probe A and probe B, S2b is the fluorescence intensity of probe A and probe B

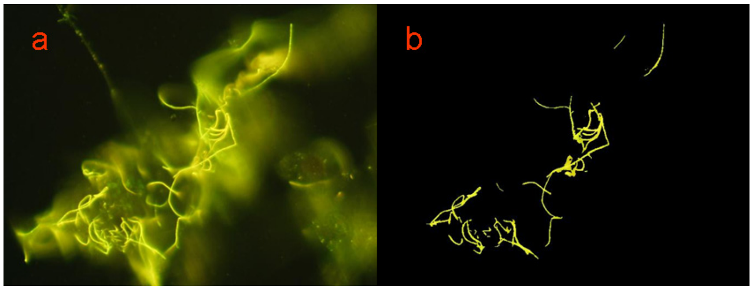

Figure S3. Example images analysis for quantification of the mean fluorescence intensity of *M. parvicella*. (**a**) The fluorescent inverted microscope (OLYMPUS-IX71) images of labeling *M. parvicella* in situ by the novel probe A; (**b**) The same visualization of *M. parvicella* as in a, in addition to the filamentous, other part is invisible, used to calculate the mean fluorescence intensity of *M. parvicella* (IOD/Area) inside the floc are clearly quantified.
